# Internists‘ career choice towards primary care: a cross-sectional survey

**DOI:** 10.1186/s12875-017-0624-2

**Published:** 2017-04-05

**Authors:** Nathalie Scherz, Stefan Markun, Vera Aemissegger, Thomas Rosemann, Ryan Tandjung

**Affiliations:** 1 Institute of Primary Care, University Hospital of Zurich, University of Zurich, Pestalozzistrasse 24, 8091 Zurich, Switzerland; 2 Arud Centres for Addiction Medicine, Zurich, Switzerland; 3 Federal Office of Public Health, Bern, Switzerland

**Keywords:** Medical education, career choice, Primary care, Survey research, Workforce

## Abstract

**Background:**

Swiss primary care (PC) is facing workforce shortage. Up to 2011 this workforce was supplied by two board certifications: general medicine and internal medicine. To strengthen them against subspecialties, they were unified into one: general internal medicine. However, since unification general practitioners’ career options are no longer restrained by early commitment to PC. This may lead to a decrease of future primary care physicians (PCPs).

**Methods:**

To gain insights in timing and factors influencing career choice of internists, we addressed a cross sectional survey to all board certified internists in the years 2000–2010 (*n* = 1462). Main measures were: final career choice (PCPs, hospital internists or subspecialists), timing and factors influencing career choice, and attractiveness of PCP career during medical school and residency.

**Results:**

Response rate was 53.2%, 44.8% were female and median age was 45 years old. Final career choice was PCP for 39.1% of participants, 15.0% chose to become hospital internists, 41.8% became subspecialists and 4.0% other. Timing of career choice significantly differed between groups. Most of the subspecialists have chosen their career during residency (65.3%), while only 21.9% of the PCPs chose during residency. Work experience in an academic hospital was negatively associated with becoming PCP (*P* < 0.001). Family influence on career choice was more frequently reported among PCPs and chiefs’ influence more reported among non-PCPs (*P* < 0.001). Fifty-nine percent of the participants considered a career as PCP to be attractive during medical school, this proportion decreased over time.

**Conclusions:**

Timing of career choice of PCPs and subspecialists strongly differed. PCPs opted late for their career and potentially modifiable external factors seem to contribute to their decision. This stresses the importance of fostering attractiveness of PC during medical school as well as during and after residency and of tailored residency positions for future PCPs in the hospital-dominated new general internal medicine training.

**Electronic supplementary material:**

The online version of this article (doi:10.1186/s12875-017-0624-2) contains supplementary material, which is available to authorized users.

## Background

As in many countries, primary care in Switzerland is challenged by a shortage of workforce and several negative prognostic factors are found when evidence across different biographical stages of physicians is looked at: during medical school, primary care has low attractiveness as a career choice [1–4]. For career choice, however, residency was found to be more determining than medical school, but attractiveness of primary care even decreases during residency [5–7]. Ultimately an important proportion of residents initially aiming at a career in primary care changes its goal during residency [1, 8, 9]. Thus, spontaneous amelioration of the workforce shortage in primary care seems unlikely.

In Switzerland, up to 2010 two different board certifications contributed to primary care workforce: the first being internal medicine with a mainly hospital based residency which also served as basis for subspecialties in internal medicine and the second being general medicine.

Both board certifications required 5 years of postgraduate medical training. There was no nationally coordinated structured curriculum, neither for future PCP nor for hospitalists. The residents designed their curriculum themselves by applying for different residency positions, mainly in hospitals. After 5 years and an examination, they submitted their completed curriculum to the institute for medical postgraduate education [10]. The institute reviewed if the criteria for the board certification were fulfilled. Board certification in general medicine required residency in- and outpatient internal medicine, surgery and different possible rotations like for example pediatrics, gynecology, psychiatry. Those board certified generalists were most likely to join primary care workforce [5, 11].

Conversely board certified internists worked mainly in internal medicine in hospital and performed rotations in internal medicine subspecialties (for example, cardiology, nephrology, angiology, endocrinology etc.) Thus they became versatile physicians qualifying for many different careers: In Switzerland internists are working as hospitalists, subspecialists and also as primary care physicians (PCPs). Therefore, residents in internal medicine are not necessarily committed to a specific career in medicine. While some residents in internal medicine clearly aim at a particular career, others may initially choose internal medicine because they thereby remain open to a broad variety of career opportunities even after board certification. Repeated surveys in Switzerland and in the US have shown that residents in internal medicine increasingly tend to opt for subspecialties and are overall inconstant in their preferences [8, 12–14].

In contrast to regulations in other countries, vocational trainings in Swiss primary care have not been mandatory for future PCPs. Between 2000 and 2010 only 30–60% of the candidates made such a vocational training [11, 15].

Countries like the Netherlands, United Kingdom, and Denmark, could address workforce shortage creating centralized dedicated primary care curricula. Most central European Countries launched similar programs. [16] Australia set incentives and programs for rural primary care workforce [17]. Another pathway was chosen In Switzerland: to counter the trend towards more sub-specialization and strengthen general internal medicine against subspecialties, the board certification in general medicine and internal medicine were merged into a single certification of “general internal medicine” in 2011. Residents are still able to design a curriculum including in- an outpatient internal medicine, subspecialties and other specialties such as surgery, pediatrics, gynecology, psychiatry in accordance to their interest and goal. Vocational training in primary care is encouraged for ongoing primary care physicians [18–20]. The effect on this merger of board certifications, however, is unknown and it may even result in a further decline of primary care workforce. Indeed, with the new certification, there will no longer be a group of general medicine practitioners that commit early to a career in primary care. Given the versatility of general internists and their broad opportunities a decrease of future PCPs might occur. We therefore aimed to gain further insights on timing of final career choice of internists and on influencing factors. We retrospectively assessed attractiveness of primary care during medical school and internal medicine residency.

## Methods

### Study design

The study is a cross-sectional survey. All physicians in the target population were invited to participate by a postal invitation letter containing a paper version of the questionnaire in either French or German language. Additionally, a link was provided leading to an identical online questionnaire. An identification code assured that only a single entry per participant was considered in the analysis. Five weeks after the first postal letter, a second postal letter was sent to non-respondents only. Both preliminary versions of the French as well as the German questionnaire were pre-tested with 15 physicians from the study population.

### Study population

All physicians who received a Swiss board certification in internal medicine in the years 2000 to 2010 were eligible. Exclusion criterion was a missing answer to the primary outcome question about final career choice. The addresses were provided by the Swiss Medical Association, FMH.

### Questionnaire

To gain insights on factors potentially influencing the career choice of medical students and residents, we conducted a literature search with the scope of general medicine, career planning and specialty choice to compose the questionnaire.

Inclusion criteria were verified with questions about years and types of board certifications.

Baseline characteristics: To characterize the study population, we included questions about demographic characteristics (sex, current age) education (age at end of medical school, place of graduation) and residency (PCP vocational training, residency at academic hospital, months of outpatient work) and current work situation (part-time, leading position, employed vs self-employed, setting, working half-days a week).

Primary outcome: The primary outcome final career choice was defined as the participants’ answer to the question “What will be your main lifetime activity”. Possible answers were: “PCP”, “hospital internist”, “subspecialists” and “other”. If the box “other” was ticked, we reclassified the participants based on their free-text answer. We reclassified them as PCP if they did not have a second board certification and currently worked in a practice. We reclassified them as “subspecialists” if they indicated a second board certification and as “hospital internist” if they were working in hospital without a second board certification. Those who were not working with patients or who did not fulfill one of the categories were classified as “others”. As our focus was PCP as a career choice, we categorized respondents as PCP versus non-PCP to compare the association of influencing factors with final career choice. Non-PCP included respondents with answers: hospital internists, subspecialists, and other career choices.

Secondary outcomes and influencing factors: To compare timing of final career choice over categories we asked “When did you decide about your main lifetime activity?” with a multiple choice answer. To investigate factors associated with the final career choice we asked about perceived influence of participants’ peers on final career choice, availability of resident positions and practice moratorium (i.e., a federal law restricting the opening of private practices in Switzerland from 2002 to 2011).

Finally we explored (non-mutually-exclusive) attractiveness of following careers: “being an internist without further subspecialty”, “being a specialist”, “working in a practice” and “working in a hospital” at different biographical time intervals: “during medical school”, “during internal medicine residency” and “final career choice”. We categorized a career in primary care to be perceived as attractive if participants rated “working in a practice” and “being an internist without further subspecialty” as attractive within the same biographical time interval.

### Statistical analyses

We summarized data using counts and proportions for categorical variables and median and interquartile range for continuous variables as all of them were all not normally distributed. Participants were grouped into PCPs and non-PCPs according to primary outcome response. Group comparisons were performed using chi-squared test defining a *P* < 0.05 as statistically significant between-group difference. Data analysis was performed using R Statistics (version 3.2.0).

## Results

Out of 3287 questionnaires 1749 were returned (response rate 53.2%). Although being in the provided address list, 266 were not board certified in internal medicine or not in the years 2000 to 2010 and thus were not included. Missing answer to the primary outcome question led to exclusion of 21 participants. Finally, 1462 answers were considered in the analysis: 1024 German, 390 French and 48 Italian (See Flow-chart in Additional file 1). Overall completeness of responses was >95% for all questions. Participants’ median age was 45 years (IQR: 42–49), 44.8% of them were female and 17.6% had finished medical school outside of Switzerland. The median number of working days per week was 5 (IQR: 3.5–5) with 47.3% of the participants working part time, 40.0% self-employed and 47.7% having a leading position.

### Primary outcome

When asked about their final career choice: 573 (39.2%) responded PCPs, 219 (15.0%) hospital internists, 611(41.8%) subspecialists and 59 (4.0%) mentioned another career choice. The baseline characteristics categorized by final career choice are presented in Table 1.

**Table 1:** Baseline characteristics

	PCPs		Hospital internists		Subspecialists		Others	
	*n*	%	*n*	%	*n*	%	*n*	%
Total	573	100	219	100	611	100	59	100
Female	311	54.3	109	49.8	203	33.2	32	54.2
Education and residency								
Swiss medical diploma	479	83.6	185	84.5	490	80.2	50	84.7
PCP vocational training	161	28.1	34	15.5	79	12.9	9	15.3
residency in an academic hospital	350	61.1	166	75.8	449	73.5	44	74.6
Current work situation								
Part time	372	64.9	85	38.8	196	32.1	26	44.1
Leading position	174	30.4	156	71.2	341	55.8	26	44.1
Employed	156	27.2	210	95.9	448	73.3	47	79.7
Setting								
Practice	527	92.0	6	2.7	189	30.9	16	27.1
Hospital	19	3.3	197	90.0	351	57.4	16	27.1
Outpatient clinic	13	2.3	10	4.6	52	8.5	4	6.8
Nursing home	4	0.7	2	0.9	4	0.7	1	1.7
Other^a^	4	0.7	2	0.9	5	0.7	21	35.6
	Median	IQR	Median	IQR	Median	IQR	Median	IQR
Age	46	42–51	45	42–49	44	41–47	45	43–49
Education and residency								
Age at end of med. school	27	26–28	26	26–28	26	26–27	27	26–28
Months of outpatient work	12	9–24	14	10–24	12	9–18	12	6–17.25
Current work situation								
Working half-days a week	9	6–10	10	7–11	10	8–11	10	6.75–10

^a^Including: administration, research & teaching, insurance, company

### Timing of career choice

**Fig. 1:**
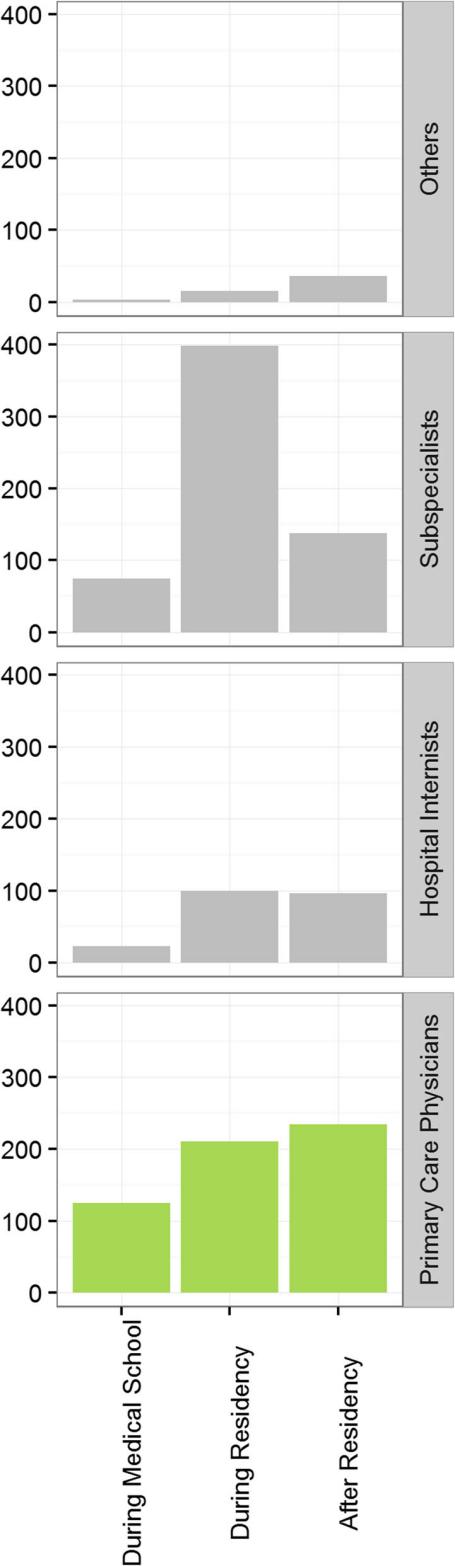
Timing of career choice

Timing of final career choice significantly differed between different groups (Fig. 1, *P* < 0.001). Most of the subspecialists have chosen their career during residency (65.3%) whereas only 37.0% of the PCPs have chosen it during that time. Conversely, more PCPs than subspecialists chose their career during medical school (21.9% versus 12.1%) and after board certification (41.1% versus 22.6%).

### Factors influencing career choice

**Fig. 2:**
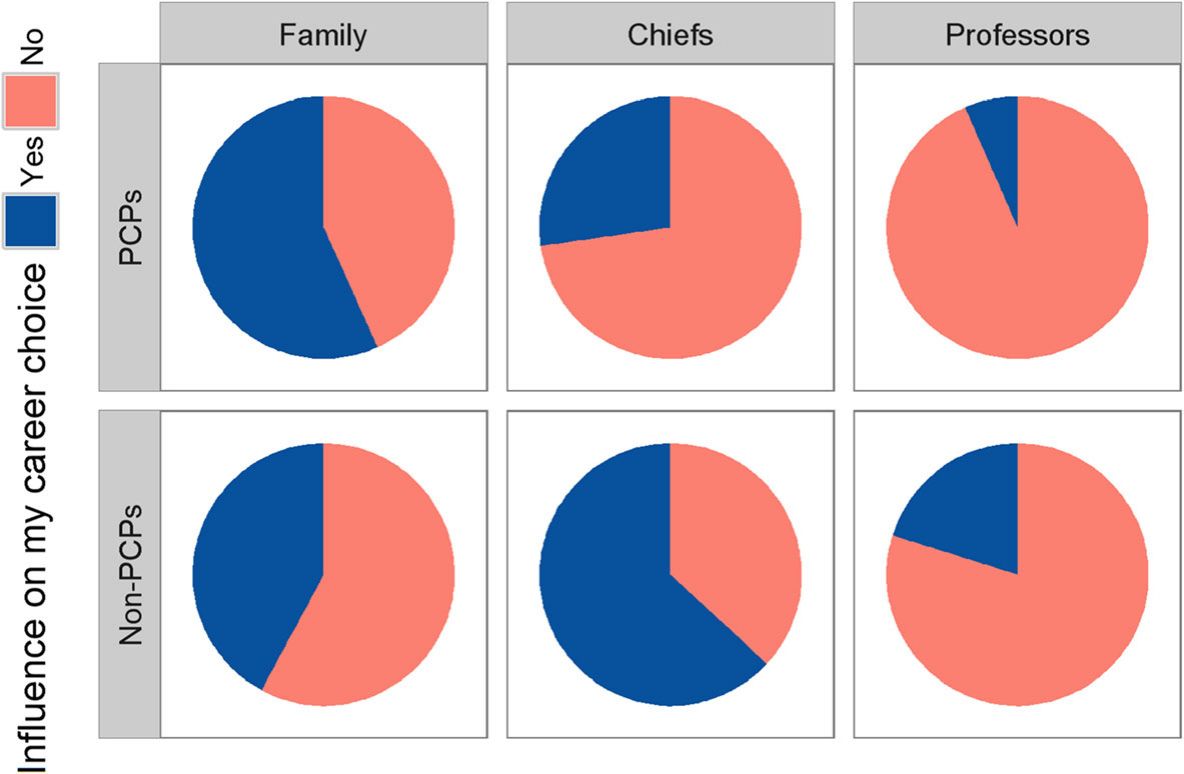
Influence of peers on career choice

Significantly more non-PCPs than PCPs felt that former chiefs (62.9% vs 26.9%, *P* < 0.001) and professors (20.0% vs 6.5%, *P* < 0.001) influenced their career choices. On the other hand, more PCPs reported influence from their family on their career choice (56.5% versus 42.0%, *P* < 0.001, Fig. 2).

Overall 25.6% of the PCPs and 24.2% of the hospital internists reported that the availability of residency positions kept them away from a sub-specialization. When asked about the influence of practice moratorium, 8.5% of the participants reported that it influenced their career choice. Among those 3.8% of the PCPs reported that it kept them from becoming a subspecialists; 8.7% of the subspecialists and 10.4% of hospital internists reported that it kept them from becoming a PCP.

Overall, a minority of the participants has completed a vocational training in primary care. More PCP than non-PCP reported having completed such a training (28.1% vs 13.7%, *P* < 0.001) and more non-PCP completed a residency in an academic hospital (38.2% vs 25.8% *P* < 0.001).

### Attractiveness of a career as a PCP

**Fig. 3:**
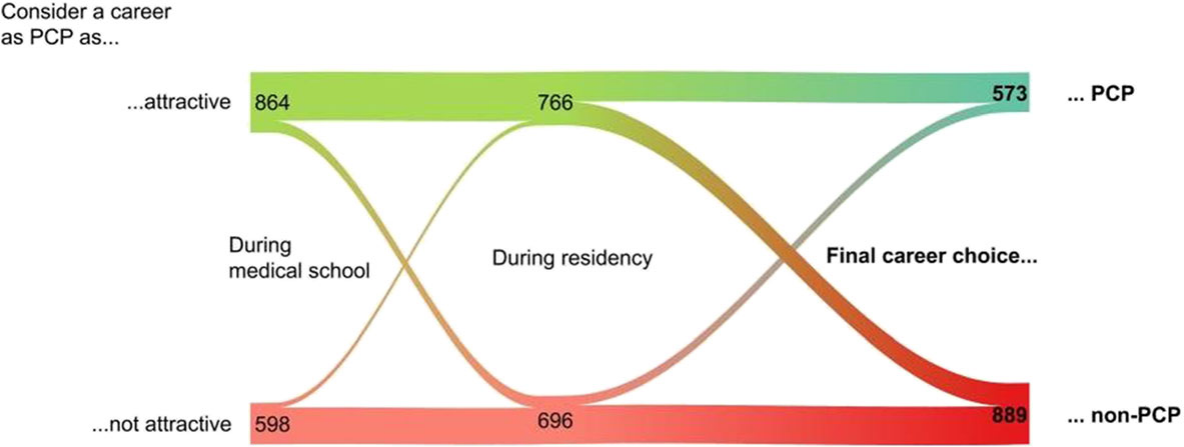
Attractiveness of a career as PCP during medical school and residency and final career choice

The perceived attractiveness of a career as a PCP changed importantly over time: 59.1% of the participants (*n* = 864) considered a career as a PCP as attractive during medical school. Until residency, there was a net decrease of 98 (52.4%) perceiving PC as attractive: A career as a PCP lost attractiveness for *n* = 169; gained attractiveness for *n* = 71. Later on, of the 766 considering a career as PCP to be attractive during residency, 333 finally chose a non-PCP career (Fig. 3).

## Discussion

We aimed at investigating timing of career choice and factors influencing choice of internists and found that those strongly differed between internists choosing to become PCPs compared to those opting for other career choices. While most of the subspecialists took their decision on future career during residency in internal medicine, more PCPs chose their career after residency. Influence of peers’ opinions also significantly differed between PCPs and non-PCPs, with non-PCPs reporting more influence of chiefs and professors and PCPs reporting more influence of family on their career choice. Half of physicians perceiving a career in primary care as attractive during residency finally opted for different career choices.

Our results complement a study made on a sample of PCPs with a board certification in general medicine: for general practitioners, residency was shown to be the most important time period for the decision to become PCPs; 53.7% of the general medicine practitioners decided during that time and overall 95.3% of them decided before board certification [5]. These numbers clearly differ from our findings in the group of PCPs with a certification in internal medicine where only 58.9% had committed to their PCP career before board certification. In our study PCPs perceived a higher influence for their career decision of their families than non-PCPs. This confirms similar results in other settings [21–24]. Meanwhile, PCPs reported less influence of chiefs and professors in their career decision. Similarly, lack of role model and “badmouthing” of PC has been shown to reduce attractiveness of PC among medical students [25, 26].

Considering Swiss healthcare’s overall shortage of physicians, the pool of residents in general internal medicine is an attractive source of workforce for almost all non-surgical medical subspecialties. Given the circumstance that general internal medicine board certification requires hospital experience but no experience in primary care, hospital careers or subspecializations simply have substantially higher odds to present themselves attractively to residents. Meanwhile, primary care remains unexperienced and unseen by most internists, even if theoretically considered to be attractive along with other possibilities. This situation might result into increasing number of undecided residents getting offered a subspecialization and to decreasing interest for a career as PCP during residency as shown in our and former studies [8, 9, 12]. The steadier primary care workforce in countries without hospital-based primary care training support this hypothesis as well [15].

We observed that during and after medical school the majority of future internists perceived a career in primary care as an attractive option. Overall the attractiveness of a career as a PCP suffered a net decrease over the career timeline: an important part of them shifts away from a PCP career when simultaneously less shift toward this career choice. For many internists, primary care rather seems to be one option in direct competition with all other career possibilities, than a specific career goal. Therefore, residents interested in primary care are vulnerable to reorientation: to a relevant extent PCPs declared to be influenced by external factors such as the availability of residency positions or the restriction to open new practices. With rising numbers of subspecialty residencies, this might contribute to the overall tendency towards more subspecialization.

Our study further showed that internists finally working as PCPs completed more primary care vocational training than non-PCPs. Still a considerable proportion of subspecialists and hospital internists had a primary care vocational training as well. Studies have shown that residents with a specific primary care residency can also be discouraged from pursuing their career goal when unsatisfying experiences are made [7, 27]. This scenario may have occurred in some of the 15.5% of hospital internists and 12.9% of subspecialists who completed a vocational training in primary care.

### Strength and limitations

Our study has potential limitations: First the cross-sectional design based on retrospective career questions is subjected to recall bias considering influencing factors and timing of the career choice. This limitation could be only addressed by prospective cohort studies starting at medical school. However, we showed that the timeline between medical school and final choice is long, so that the follow-up must be planed over more than 10 years in order to capture the late decision concerning a very important amount of the physicians. Second our questionnaire contained no comprehensive set of all possible factor influencing the career choice, as those are complex, partly conscious and sometime very individual. Strengths are the successful sampling of the study with a high response rate of the survey and across internists graduating during a broad time interval ranging from 2000 to 2010. The presented spectrum of influencing factors is therefore less likely to be influenced by single events.

### Implication for practice

In Switzerland, since the unification of internal and general medicine, all residents aiming to become general internists – independently whether they aim to become PCPs early or not – are qualifying for many different careers. Considering that external factors (including chiefs’ opinion, availability of residency positions) are influencing their career choice during residency, the new combined board certification in general internal medicine might even decrease the number of PCPs [8, 14, 21]. Attention must therefore be taken to keep motivation high for future PCPs. Residency has been shown to be crucial concerning career choice leading to primary care. Besides attractive career paths with structured residency programs, role models as well as support by chief and academic staff are important, which can be supported by academic institutes of primary care and mentoring programs. [28–30] If these measures fail, the number of future PCPs might decline even more despite the increasing number of certified general internists [31].

## Conclusion

Career choice timing and influence factors of PCPs and subspecialists strongly differ. Internists working as PCPs decide late for a career in primary care and remain vulnerable to external factors, including the availability of residency positions or issues influencing future working conditions. In a very flexible system such as the one in Switzerland, where residents are able to change career orientation at any time of their medical education, continuous investments to keep attractiveness of primary care high during medical school and during and after residency.

## Additional file

Additional file 1: Figure S1. Inclusion flow-chart. (PDF 8 kb)

## Abbreviations

PC: Primary care
PCP: Primary care physician
