# Supplementary material for: Internists‘ career choice towards primary care: a cross-sectional survey
Source: BMC Fam Pract. 2017 Apr 5;18:52. doi: 10.1186/s12875-017-0624-2 (PMC5382387; doi:10.1186/s12875-017-0624-2)
Supplement: Additional file 1: Figure S1. — Inclusion flow-chart. (PDF 8 kb) [file 12875_2017_624_MOESM1_ESM.pdf]

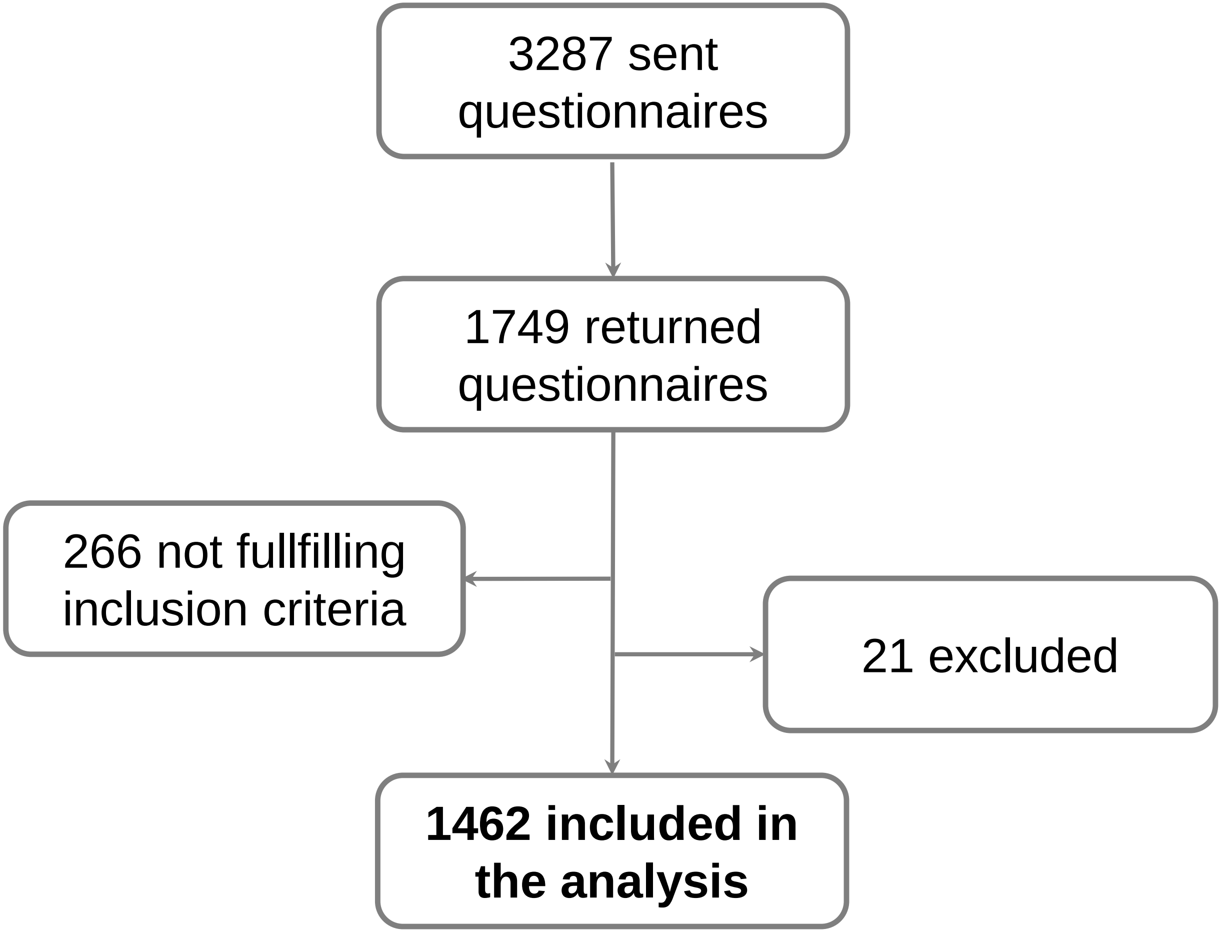
3287 sent questionnaires
1749 returned questionnaires
266 not fullfilling inclusion criteria
21 excluded
1462 included in the analysis
